# Ambient AI Scribe Technology and Perceived Documentation Efficiency Among Surgeons: Cross-Sectional Study

**DOI:** 10.2196/89329

**Published:** 2026-10-13

**Authors:** Sophia Luo, Katlyn McKay, Tatiana Zabaleta, Jeffrey S Upperman, Jamie R Robinson

**Affiliations:** 1 Department of Pediatric Surgery, Vanderbilt University Medical Center, Monroe Carell Jr. Children's Hospital, 2200 Children's Way, Nashville, TN, 37232, United States, 1 919 935 1339; 2 Section of Surgical Sciences, Vanderbilt University Medical Center, Nashville, TN, United States; 3 Department of Biomedical Informatics, Vanderbilt University Medical Center, Nashville, TN, United States

**Keywords:** ambient AI, clinical documentation, Dragon Ambient Experience Copilot, DAX Copilot, surgeons, physician burnout, documentation efficiency, documentation practices, artificial intelligence, AI

## Abstract

**Background:**

Clinical documentation demands a significant share of surgeons’ time and can contribute to documentation-related burden and burnout. While electronic health records (EHRs) offer accessibility and completeness, they also impose a substantial documentation burden. Ambient AI scribe solutions aim to reduce this burden by capturing patient-clinician dialogue in real time to automate clinical documentation. However, their specific impact on surgical practice remains under-studied.

**Objective:**

We aimed to evaluate surgeons’ perceptions of documentation efficiency, documentation timeliness, and burnout following implementation of an ambient AI documentation tool within the EHR during outpatient clinical encounters.

**Methods:**

We conducted a single-center cross-sectional survey of all surgical faculty members at a tertiary care academic institution 3 months after institutional implementation of the ambient AI-powered clinical documentation tool Dragon Ambient Experience (DAX) Copilot. A total of 368 faculty surgeons representing 12 surgical subspecialties were invited to participate. Participants were asked to retrospectively assess their baseline perceptions of documentation burden and subsequently report perceived changes following DAX Copilot implementation using standardized Likert-scale survey items. Descriptive analyses were performed among active DAX Copilot users. Open-ended responses were analyzed using practical thematic analysis.

**Results:**

The survey was completed by 28.5% (105/368) of the invited surgeons, with 38.1% (40/105) of participants reporting active DAX Copilot use. Overall, 77.5% (31/40) of users reported some or significant improvement in perceived documentation efficiency. Of those who reported a negative or neutral baseline perception of documentation time, 75.8% (25/33) reported an improved perception of efficiency following implementation of the ambient AI tool, whereas 85.7% (6/7) of surgeons with positive baseline perceptions of documentation time also reported further improvement. Improvements in perceived documentation timeliness were reported by 70.4% (19/27) of surgeons who previously reported negative or neutral baseline perceptions of timeliness and 76.9% (10/13) of surgeons with positive baseline perceptions. Additionally, 72.5% (29/40) of respondents reported enhanced documentation accuracy, detail, and consistency after DAX Copilot implementation. Improvements in self-reported burnout levels were reported less frequently, with 59.0% (23/39) of respondents reporting some or significant improvement, including 63.0% (17/27) with negative or neutral baseline perceptions of burnout. Qualitative feedback highlighted perceived improvements in documentation quality while also identifying current limitations of the technology, including challenges related to EHR integration, device compatibility, and customization.

**Conclusions:**

In this single-center survey of surgeons using DAX Copilot in their outpatient clinical practice, use of ambient AI scribe technology was associated with improved perceptions of documentation efficiency and timeliness. These findings suggest that ambient AI tools may improve surgeons’ perceived documentation experience in outpatient surgical care.

## Introduction

Clinical documentation, while essential, has become an increasing source of strain for physicians, particularly with the rise and rapid expansion of electronic health records (EHRs) over the past few decades. Increased time expenditure on documentation has been shown to increase the odds of burnout among health care providers, which in turn has been linked to worse patient outcomes and increased medical errors [1-3]. For surgeons specifically, reported burnout rates range from 30% to 45%, with commonly cited contributing factors including limited control over time, challenges with work-life balance, and documentation burden [4,5]. The literature has demonstrated inefficiencies in the documentation process and massive time investments requiring up to 73.3 twenty-four–hour days of total annual documentation time among a group of surgeons within a trauma setting [6]. In one study of surgical residents specifically, survey participants reported spending at least 30% of their 80-hour work week using the EHR [7]. As concerns regarding surgeon shortages and workforce attrition continue to grow, interventions that reduce administrative burden may play an increasingly important role not only in supporting physician well-being but also in promoting workforce sustainability, retention, and long-term access to surgical care.

Emerging technologies such as ambient AI scribes offer promising strategies to reduce documentation burden that traditional approaches such as medical scribes, workflow modifications, educational initiatives, technological upgrades, and clinical documentation specialists have only partially addressed [8,9]. Ambient AI agents listen to clinician-patient conversations and use generative AI to draft notes as a digital scribe. Early evaluations of ambient AI documentation technologies have demonstrated improvements in documentation efficiency, reductions in documentation and after-hours EHR time, and favorable clinician perceptions across ambulatory practice settings [10-15]. For primary care providers specifically, documentation time was reduced by 28.8% [16]. In addition to improved time to documentation completion, ambient AI has been shown to decrease burnout rates across clinicians in select studies [17]. In one analysis performed in 2025, the proportion of clinicians reporting burnout decreased from 52.6% to 30.7% over a 42-day study period. Furthermore, users of ambient AI noted higher rates of documentation-related well-being [10,15]. However, evidence has been mixed across the literature regarding burnout reduction, with an additional study of 110 primary care providers showing no change in burnout scores after implementation [16].

Despite promising data from primary care and other ambulatory specialties, evidence evaluating ambient AI documentation technologies specifically in surgical practice remains limited. Although recent studies have included surgeons within broader ambulatory clinical cohorts, few investigations have focused specifically on surgeons across multiple surgical subspecialties, and relatively little is known about surgeons’ perceptions of ambient AI documentation technologies [18-21]. Furthermore, surgical outpatient encounters differ structurally from those in previously studied specialties as they are often centered on consultation, procedure planning, and operative decision-making, with documentation requirements encompassing informed consent, perioperative planning, and multidisciplinary coordination. Billing for outpatient surgical encounters also differs greatly from that for most nonprocedural specialties as it is typically dependent upon medical decision-making being adequately documented rather than on a time-based billing model. Consequently, the extent to which the benefits of these technologies observed in broader ambulatory populations translate to outpatient surgical practice remains uncertain. An ambient AI-powered clinical documentation tool, Dragon Ambient Experience (DAX) Copilot, embedded within the EHR was implemented at our institution and made available to all surgeons for their outpatient clinical encounters. We sought to understand whether the implementation of ambient AI for surgeons would improve perceived documentation efficiency and burnout.

## Methods

### Overview

We evaluated the early implementation of DAX Copilot across surgical subspecialties through a cross-sectional survey of all surgical faculty members at Vanderbilt University Medical Center. A total of 368 faculty surgeons representing 12 surgical subspecialties were identified and invited to participate. Surgical trainees, including residents and fellows, were not eligible for use during the initial rollout of the AI scribe and were therefore not included in the present study. Surgical subspecialties included cardiac surgery, otolaryngology, neurological surgery, ophthalmology, oral and maxillofacial surgery, orthopedics, pediatric surgery, plastic surgery, general surgery (including trauma, colorectal surgery, minimally invasive surgery, liver or kidney transplant, surgical oncology, and other related areas), thoracic surgery, urology, and vascular surgery.

To ensure successful adoption, surgeons received comprehensive onboarding and training in the months prior to DAX Copilot integration into outpatient workflows on January 15, 2025. Prior to full deployment, surgeons completed onboarding that included self-service training materials, in-person and virtual guided sessions, and one-to-one support as needed to ensure familiarity with the mobile EHR app, patient consent workflow, and the editing of AI-generated notes. DAX Copilot is a HIPAA (Health Insurance Portability and Accountability Act)-compliant generative clinical voice technology facilitated by AI and owned by Nuance Communications, a Microsoft company. This emergent health technology is meant to reduce the administrative burden on health care providers by using a large language model to document and transcribe patient-provider interactions in real time. DAX Copilot is integrated into the EHR and uses conversational generative AI to automatically and instantaneously draft clinical notes after a patient visit. With patient consent, clinicians can initiate recording of the clinical encounter through the mobile EHR app on their personal iOS devices. DAX Copilot passively captures the clinician-patient conversation in real time. Upon completion of the visit, the audio recording is securely uploaded to a cloud-based service, where it is transcribed and transformed into a draft clinical note. The draft note is then returned to the EHR for clinician review and editing, after which the finalized note is saved directly into the patient’s chart. Adoption of the technology was voluntary, and surgeons were permitted to use their own discretion regarding whether to incorporate DAX Copilot into their clinical workflow.

### Outcome Assessment

We assessed surgeons’ experiences with DAX Copilot 3 months after implementation throughout April 2025 by distributing a REDCap survey (Vanderbilt University) to all surgical faculty, with a focus on burnout and documentation practices. The survey collected qualitative and quantitative data regarding surgeons’ experiences with the AI tool. Assessed domains included perceived burnout, time spent on documentation, and timeliness of documentation before and after implementation. Because the survey was administered only after DAX Copilot implementation, respondents were first asked to retrospectively assess their baseline perceptions prior to implementation and were then asked to evaluate the perceived impact of DAX Copilot on each domain. Responses to both retrospective baseline and postimplementation questions were captured using standardized 5-point Likert scales. Additional postimplementation questions evaluated surgeons’ perceptions of patient care, along with a single composite item assessing the perceived impact of DAX Copilot on documentation accuracy, detail, and consistency. While the survey items were not derived from formally validated assessment instruments, they were informed by prior literature evaluating health care provider burnout and documentation burden. An optional open-ended survey question was included to allow respondents to provide additional feedback regarding their experience with DAX Copilot, including perceived benefits, limitations, or implementation challenges not fully captured by the structured survey items. Open-ended responses were analyzed using practical thematic analysis. The first author (SL) independently reviewed all responses, identified recurring themes, and grouped the feedback into categories reflecting perceived benefits and opportunities for improvement. Themes were reviewed by a second author (JRR) for accuracy assurance; however, independent coding and formal assessment of interrater reliability were not performed. Representative quotations were selected to illustrate the themes that were identified.

### Ethical Considerations

This study was reviewed and approved by the Vanderbilt University Medical Center Institutional Review Board (250238). This study was determined to meet all applicable ethical requirements for research involving human participants. Participation was voluntary. An electronic informed consent statement was presented at the beginning of the survey, and participants indicated their consent by proceeding to the survey. Participants could discontinue participation at any time without penalty.

### Data Analysis

We analyzed surgeons’ perceptions of documentation before and after DAX Copilot implementation using descriptive statistics. Baseline characteristics were compared between active DAX users and nonusers. Categorical variables were compared using the Pearson chi-square test or Fisher exact test when expected cell counts were small. Statistical significance was defined as *P*<.05. Responses to 5-point Likert-scale questions were dichotomized for descriptive analyses. For baseline assessments, responses of “none” or “slight” for perceived burnout, “minimal” or “low” for perceived time spent on documentation, and “optimal” or “good” for perceived documentation timeliness were classified as positive baseline perceptions. All remaining responses were classified as negative or neutral baseline perceptions. For postimplementation assessments, responses indicating “significant” or “some” improvement were classified as a positive change, whereas all other responses were classified as no improvement or worsening. Analyses of survey items with missing data were based on available responses. Results are presented as frequencies and percentages for the overall cohort and stratified by baseline perception where applicable. Analyses were performed using R (version 4.5.2; R Foundation for Statistical Computing) [22].

## Results

Survey responses from a diverse range of surgical subspecialties revealed early adoption patterns and demographic trends among DAX Copilot users. Of the 368 surgical faculty members surveyed, 105 (28.5%) completed the questionnaire. At the time of the survey, 3 months after DAX Copilot implementation, 38.1% (40/105) of the respondents reported actively using the tool (Figure 1).

These 40 surgeons represented 9 distinct surgical subspecialties. Most (24/40, 60%) were male. Nearly one-quarter (9/40, 22.5%) had fewer than 5 years of practice experience, with the remainder distributed relatively evenly across longer practice durations (Table 1).

**Figure 1.**
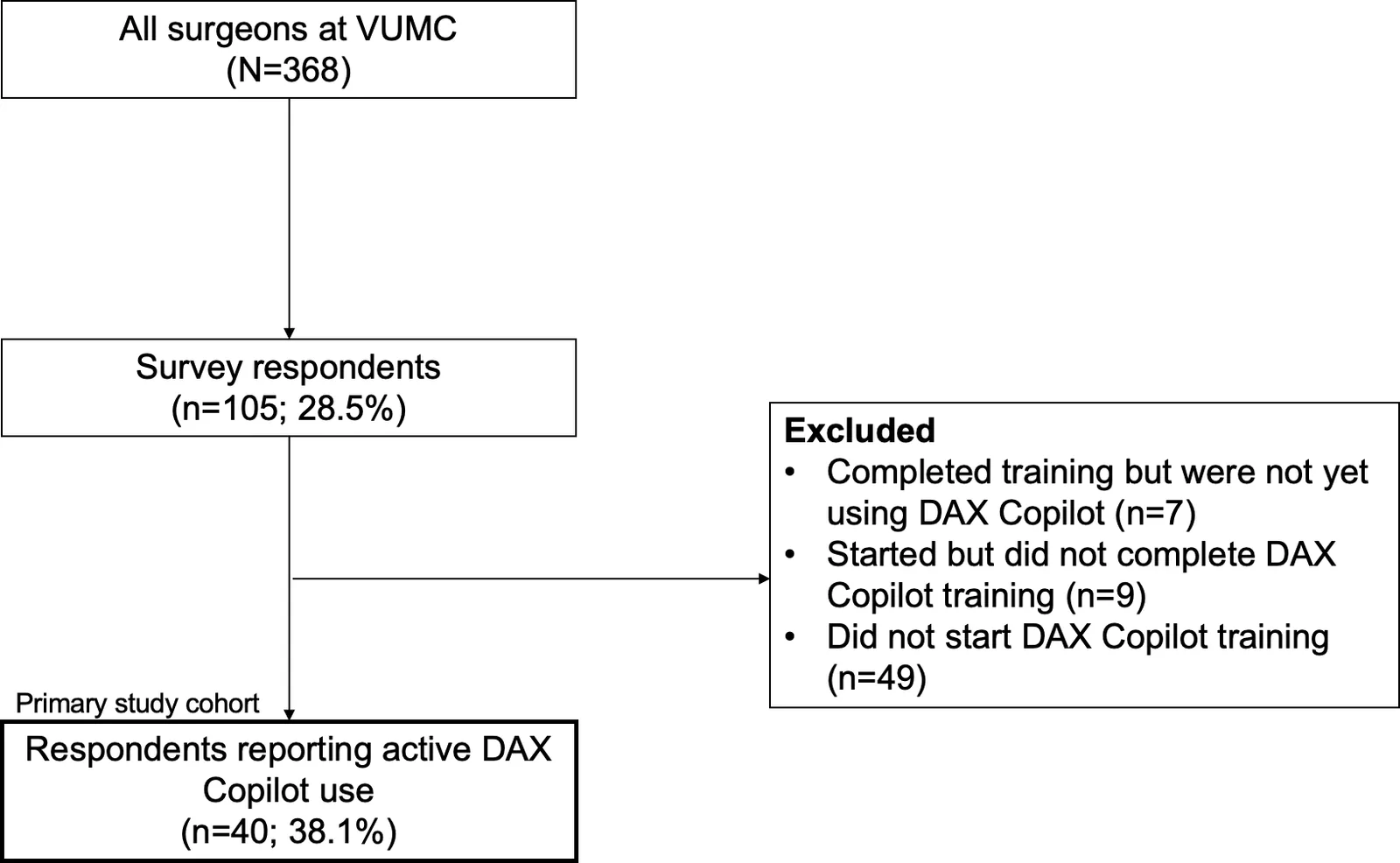
Cohort selection diagram for a single-center cross-sectional survey evaluating surgeons’ perceptions of the ambient AI documentation tool Dragon Ambient Experience (DAX) Copilot at Vanderbilt University Medical Center (VUMC) 3 months after implementation. Of 368 eligible surgeons invited to participate, 105 completed the survey. Respondents who were not actively using DAX Copilot or who had not yet started or completed training were excluded, yielding a final analytic cohort of 40 active DAX Copilot users.

**Table 1.** Baseline characteristics of respondents to a single-center cross-sectional survey evaluating use of the ambient AI tool Dragon Ambient Experience (DAX) Copilot among surgeons at Vanderbilt University Medical Center 3 months after implementation. Characteristics are compared between surgeons reporting active DAX Copilot use and respondents not actively using DAX Copilot.

Characteristics	Active DAX users (n=40), n (%)	DAX nonusers (n=65), n (%)	*P* value
Sex			.51
Female	12 (30)	17 (26.2)	
Male	24 (60)	45 (69.2)	
Prefer not to answer	4 (10)	3 (4.6)	
Specialty			.17
Cardiac surgery	0 (0)	1 (1.5)	
Otolaryngology	8 (20)	5 (7.7)	
Neurological surgery	2 (5)	3 (4.6)	
Ophthalmology	2 (5)	9 (13.8)	
Oral and maxillofacial surgery	0 (0)	2 (3.1)	
Orthopedics	6 (15)	12 (18.5)	
Pediatric surgery	4 (10)	3 (4.6)	
Plastic surgery	2 (5)	3 (4.6)	
Surgery^a^	11 (27.5)	19 (29.2)	
Thoracic surgery	0 (0)	0 (0)	
Urology	2 (5)	8 (12.3)	
Vascular surgery	3 (7.5)	0 (0)	
Experience (y)			.67
<5	9 (22.5)	13 (20)	
5-10	6 (15)	16 (24.6)	
11-15	8 (20)	8 (12.3)	
16-20	5 (12.5)	10 (15.4)	
21-25	6 (15)	6 (9.2)	
≥26	6 (15)	12 (18.5)	
Baseline perceived burnout			.51
None	5 (12.5)	8 (12.3)	
Slight	8 (20)	18 (27.7)	
Moderate	15 (37.5)	16 (24.6)	
High	8 (20)	19 (29.2)	
Very high	4 (10)	4 (6.2)	
Baseline perceived documentation time			.79
Minimal	2 (5)	4 (6.2)	
Low	5 (12.5)	12 (18.5)	
Moderate	12 (30)	24 (36.9)	
High	16 (40)	19 (29.2)	
Excessive	5 (12.5)	6 (9.2)	
Baseline perceived documentation timeliness			.11
Optimal	7 (17.5)	19 (29.2)	
Good	6 (15)	11 (16.9)	
Satisfactory	2 (5)	8 (12.3)	
Marginal	18 (45)	24 (36.9)	
Poor	7 (17.5)	3 (4.6)	

a Trauma, colorectal surgery, minimally invasive surgery, liver and kidney transplant, surgical oncology, and other adult general surgery specialties not listed separately (eg, acute care surgery and surgery critical care).

All 40 DAX Copilot users provided responses to each baseline survey item. Following DAX Copilot implementation, 77.5% (31/40) of surgeons reported some or significant improvement in perceived documentation efficiency irrespective of their baseline perceptions of documentation time. Among those with negative or neutral baseline perceptions (n=33), 75.8% (25/33) reported an improved perception of efficiency after DAX Copilot implementation, and among those with positive baseline perceptions (n=7), 85.7% (6/7) reported additional efficiency gains (Figure 2).

**Figure 2.**
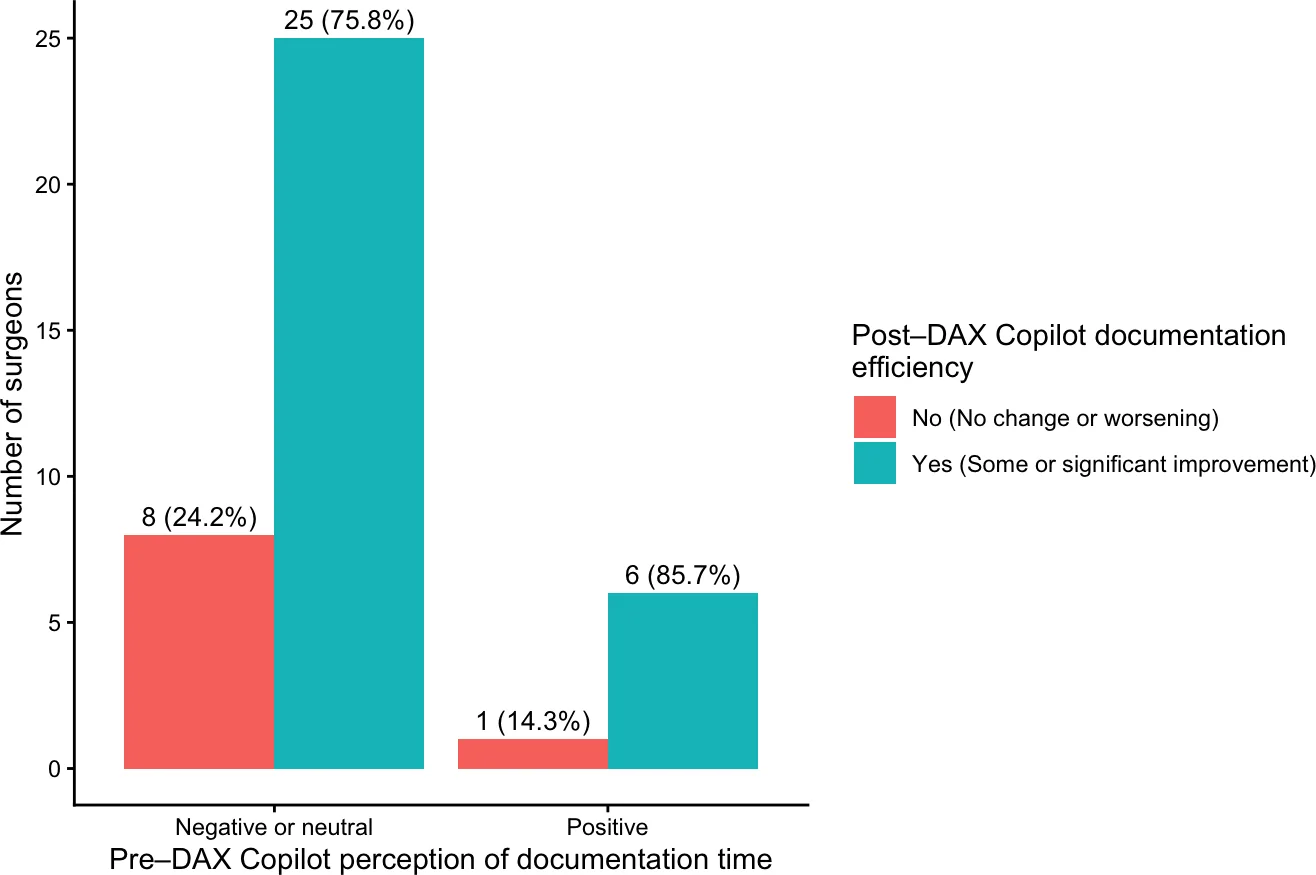
Reported improvement in perceived documentation efficiency following Dragon Ambient Experience (DAX) Copilot implementation according to baseline perceived documentation time. Baseline documentation time perceptions were characterized as negative or neutral (“excessive,” “high,” or “moderate” response options) or positive (“low” or “minimal” response options). Post–DAX Copilot documentation efficiency was characterized as either no change or worsening or some or significant improvement. Bars represent the number of respondents, with percentages reflecting the proportion of respondents within each baseline perception group.

Perceived documentation timeliness also improved after implementation. Among surgeons with negative or neutral perceptions of their documentation timeliness before DAX Copilot adoption (n=27), 70.4% (19/27) reported improvements in perceived timeliness after adopting DAX Copilot. Similarly, among those with positive baseline impressions (n=13), 76.9% (10/13) reported further improvements in timeliness (Figure 3).

When surgeons were asked specifically about their experience following use of DAX Copilot, 72.5% (29/40) reported that their perceived documentation accuracy, detail, and consistency had improved or significantly improved. Improvement in burnout was reported less frequently, with 59.0% (23/39) of respondents reporting some or significant improvement (n=1 missing response from a surgeon with a neutral baseline burnout perception). Among surgeons with negative or neutral baseline perceptions of burnout (n=27), 63.0% (17/27) reported some or significant improvement after adopting DAX Copilot.

**Figure 3.**
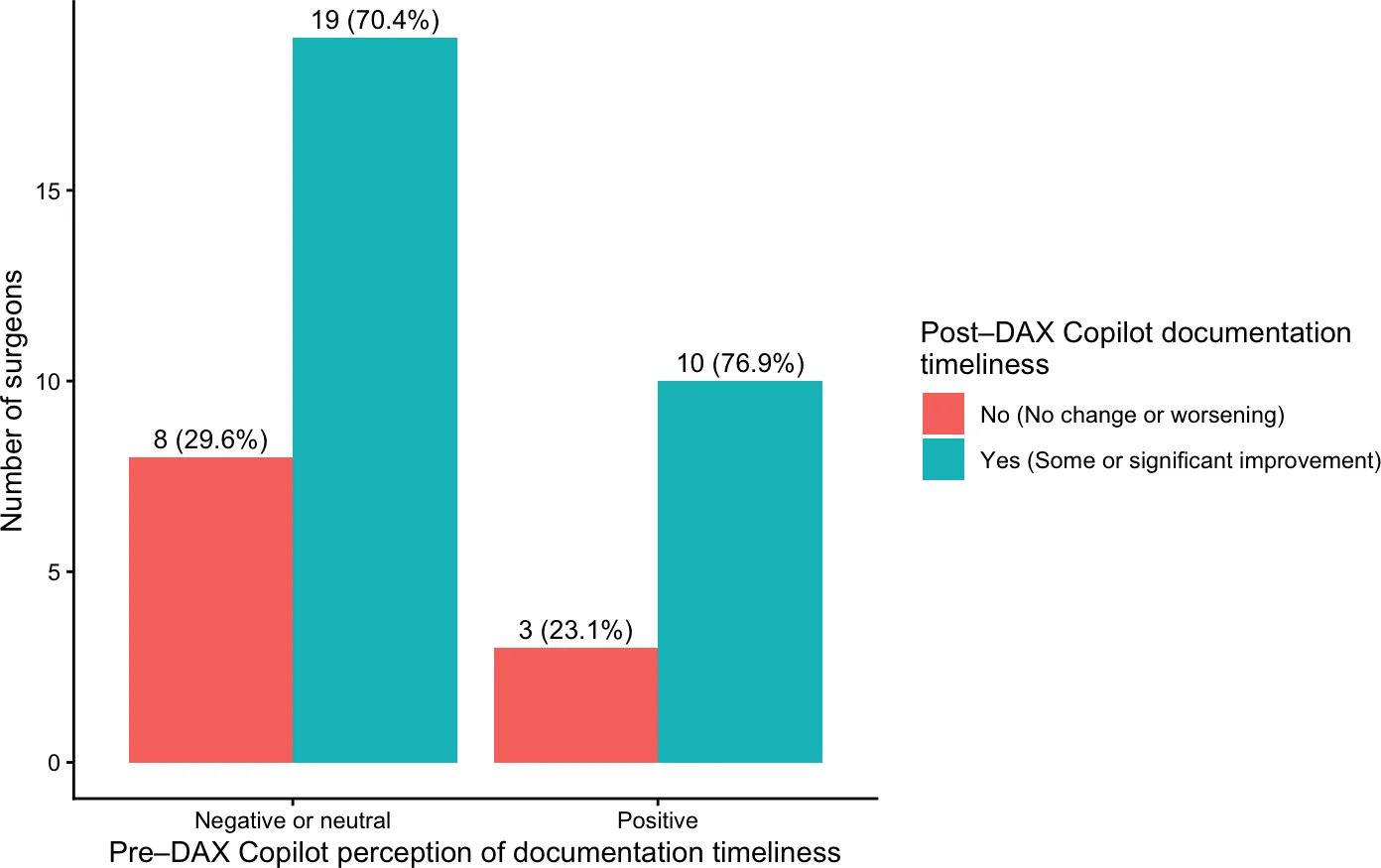
Reported improvement in perceived documentation timeliness following Dragon Ambient Experience (DAX) Copilot implementation according to baseline perceived documentation timeliness. Baseline perceptions of documentation timeliness were characterized as negative or neutral (“poor,” “marginal,” or “satisfactory” response options) or positive (“good” or “optimal” response options). Post–DAX Copilot documentation timeliness was characterized as either no change or worsening or some or significant improvement. Bars represent the number of respondents, with percentages reflecting the proportion of respondents within each baseline perception group.

Open-ended survey responses from all 105 responding surgeons, including those who had and had not used DAX Copilot, offered additional insights into DAX Copilot’s utility and opportunities for refinement (Table 2).

Surgeons reported faster note completion and improved capture of complex discussions or detailed patient histories, enabling greater focus on the patient during encounters. These benefits were most apparent during new or complex visits. However, several recurring workflow challenges emerged. Respondents noted limited EHR integration, which prevented the automated insertion of laboratory or imaging results. They also reported challenges with the use of “dot” or “smartphrases” within the Epic EHR, mechanisms that note writers often use to aid with efficiency. Others noted poor performance for follow-up visits or carried-forward notes due to the inability to automatically incorporate information from prior patient encounters. Additional concerns included accuracy limitations and the learning curve inherent to any significant technological change. Adoption was further constrained by DAX Copilot’s current iOS-only compatibility, which excluded Android users. Despite these limitations, many surgeons still recognized the technology’s potential and indicated that they planned to continue using it.

**Table 2.** Surgeons’ responses regarding the utility of Dragon Ambient Experience Copilot.

Theme	Representative surgeon comments
Perceived benefits of ambient AI	
Improved documentation efficiency	“Still do a fair amount of editing but saves a lot of time.” [User]
Improved patient engagement during visits	“Does free me up to speak to the patient without jotting down notes.” [User]
Reduced cognitive load, particularly for new or complex patients	"I can focus on the patient. It really acts as an information storage mechanism so I don’t have to remember specifics of the history and exam afterward.” [User]
Timelier documentation completion	“DAX certainly provides more timely documentation and closing of encounters.” [User]
Anticipated long-term potential	“I think in the long run this will be a very helpful technology.” [User]
Challenges and barriers to implementation	
Limited EHR^a^ integration	“If I reference a lab result or a CT scan for the patient, instead of ‘typing/generating’ text around that, it would be more beneficial for DAX to find the lab or imaging result in the EHR and copy/paste it into the note automatically.” [User]
Limited ability to customize or format progress notes	“Difficult to incorporate sections into carried forward notes and can’t customize how information is organized.” [User]
Accuracy limitations	“Doesn’t capture many critical elements to optimize billing and often misinterprets correct operation.” [User]
Training and implementation barriers	“Many of us would like to use but do not want to impede workflow while trying to integrate this tool.” [Nonuser]
Technology access limitations	“I understand that DAX is not available for Android based devices (I do not use Apple/iPhone) so it may be some time before it works into my current workflow.” [Nonuser]

a EHR: electronic health record.

## Discussion

### Principal Findings

In this study, we evaluated the early impact of an ambient AI technology integrated within the EHR (DAX Copilot) on perceived documentation efficiency and burnout among surgeons in their outpatient clinical encounters. Our findings suggest that ambient AI may improve surgeons’ perceptions of documentation processes in these settings. Overall, the implementation of DAX Copilot was associated with perceived improvements in documentation timeliness and overall efficiency, suggesting that ambient AI tools may help reduce perceived documentation burden among surgeons.

### Comparison to Prior Work

These results build on a growing body of literature demonstrating that ambient AI documentation technologies may reduce documentation burden across ambulatory practice settings. Early prospective studies on the impact of ambient AI have shown that health care providers spend less time on documentation and after-hours EHR work and that ambient AI reduces total EHR time following implementation [15,16]. More recently, a multicenter quality improvement study by Olson et al [14] reported improvements in clinician burnout, note-related cognitive task load, and time spent documenting following the adoption of ambient AI. Our findings are consistent with those of these prior reports while extending the literature by specifically evaluating surgeons across a broad range of surgical subspecialties and demonstrating perceived improvements regardless of baseline documentation perceptions. Notably, our study found that DAX Copilot improved perceived documentation efficiency and timeliness among surgeons regardless of whether their baseline perceptions of documentation were positive or negative. These findings suggest that the advantages of DAX Copilot extend beyond individuals who initially viewed documentation as inefficient, underscoring its potential as a beneficial tool that may improve documentation workflow for a broad range of users.

Open-ended user feedback revealed both enthusiasm for AI-assisted documentation and critical barriers to full integration and usability. The reported improvements in documentation quality and completeness, particularly for new or complex patients, suggest that surgeons perceived DAX Copilot as a facilitator for comprehensive documentation without compromising on the amount of information that is captured during the clinical encounter. Conversely, the limitations raised by surgeons, including limited EHR integration, suboptimal performance for follow-up visits, and restricted device compatibility, reflect key workflow barriers that must be addressed to expand the usability and maximize the value of AI-assisted transcription tools. Nonetheless, the willingness of surgeons to continue using DAX Copilot despite these challenges indicates that users perceived meaningful value in the technology and were receptive to integrating it into their clinical workflow as it continues to evolve.

Perceived improvements in burnout were reported by most respondents but occurred less frequently than improvements in documentation efficiency and timeliness. Prior evidence of burnout reduction with ambient AI use has been mixed [14,16]. Our findings suggest that while improvements in documentation may contribute to reduced burnout for some surgeons, clinical documentation likely represents only one of several factors that contribute to overall surgeon burnout.

Despite these potential benefits, important ethical and privacy considerations remain. Because these systems rely on audio capture of clinical encounters, concerns may arise regarding sensitive conversations, patient privacy, and data security. Although DAX Copilot is a HIPAA-compliant platform with enterprise-level security safeguards and encrypted EHR integration, the risk of unauthorized access or data breaches cannot be entirely eliminated. As adoption of ambient AI technologies expands, ongoing attention to patient communication, institutional oversight, and privacy safeguards will be essential to ensure responsible integration into clinical practice.

Our early-stage evaluation offers valuable insights while highlighting the need for larger studies and longer-term follow-up to assess how ambient AI technology may influence surgeon workflow. To our knowledge, this study is among the first to examine surgeons’ perceptions of ambient AI technology in outpatient surgical practice among multiple surgical subspecialties and one of the first to examine perceived improvements according to baseline documentation perceptions. We queried surgeons across a broad range of surgical subspecialties and systematically assessed their perceptions using standardized Likert-scale measures, providing an early characterization of surgeon perceptions during the initial adoption of this technology.

### Limitations

Several limitations should be acknowledged when interpreting these findings. First, while the survey response rate was moderate at 28.5% (105/368), only 40 respondents reported actively using the software and were included in the final analysis. It is thus possible that the views of all surgeons and surgical subspecialties were not fully represented by this sample. The potential for nonresponse bias should also be considered as surgeons who elected to complete the survey or actively adopted DAX Copilot may have differed systematically from nonrespondents in their documentation burden, attitudes toward ambient AI technology, or willingness to incorporate new technologies into clinical practice. Additionally, the modest sample size limits the precision and generalizability of our findings. Future multicenter studies including larger cohorts of surgeons across diverse surgical subspecialties will help improve the representativeness of these findings.

Second, this study was designed to evaluate surgeons’ early experiences during the initial implementation of DAX Copilot using a single postimplementation survey. Respondents retrospectively assessed their baseline perceptions before reporting perceived improvement following DAX Copilot implementation. Consequently, the findings may have been influenced by recall bias and response shift bias, where users who adopted the technology may have retrospectively perceived their preimplementation documentation burden more negatively, potentially resulting in an overestimation of the perceived magnitude of improvement. In addition, surgeons reporting slight or no baseline burnout had limited opportunity to report further improvement, introducing a potential floor effect that should be considered when interpreting the observed proportion of respondents reporting improvements in burnout. Future prospective longitudinal studies incorporating preimplementation baseline assessments will help better characterize the impact of ambient AI documentation technologies over time.

Third, although the survey items used in this study were informed by prior literature evaluating health care provider burnout and documentation burden, they were not formally validated, which may limit reliability and comparability across studies. Likert-scale responses were also dichotomized for descriptive analyses, reducing response granularity.

Fourth, the qualitative component of this study was intended to provide contextual insights into surgeons’ experiences with DAX Copilot. Although the identified themes were reviewed by a second author for accuracy assurance, the initial thematic analysis was conducted by a single reviewer without independent coding or formal assessment of interrater reliability. As a result, the qualitative findings may be subject to interpretive bias and should be considered exploratory.

Fifth, because this was an observational study conducted during the early implementation of DAX Copilot, we cannot exclude the possibility that concurrent changes in clinical workflows, EHR functionality, or other software enhancements influenced surgeons’ perceptions of documentation burden and efficiency. Accordingly, the observed perceptions of improvement cannot be attributed solely to DAX Copilot.

Finally, perceived utility may differ between early and late adopters of this technology or may evolve as familiarity with the technology increases over time. Notably, while DAX Copilot was only available in outpatient clinical encounters during the initial rollout phase, it is now being adopted gradually in the inpatient environment, where documentation burden persists. As adoption expands across surgical areas, specialties, and clinical settings, the perceived utility of ambient AI technologies and their potential impact on burnout may become more apparent.

### Future Directions

Future studies should prospectively evaluate ambient AI technologies using longitudinal study designs that combine clinician-reported outcomes with objective measures of documentation burden and efficiency to better characterize the impact of these technologies on documentation workflows and burnout over time. Although recent multicenter studies have demonstrated improvements in documentation burden and clinician well-being across diverse ambulatory practice settings, additional research focused on surgeons across surgical subspecialties and practice settings will help clarify the generalizability of these findings within surgical practice specifically. In addition, further research evaluating how the perceived utility of ambient AI technologies evolves with increasing user familiarity and broader implementation across outpatient and inpatient surgical settings will help clarify their long-term role in surgical documentation.

### Conclusions

In this single-center survey of surgeons using DAX Copilot in their outpatient clinical practice, use of ambient AI scribe technology was associated with perceived improvements in documentation efficiency and timeliness. These findings suggest that ambient AI documentation technology may improve surgeons’ perceived documentation experience in outpatient surgical settings.

## Abbreviations

DAX: Dragon Ambient Experience
EHR: electronic health record
HIPAA: Health Insurance Portability and Accountability Act
